# A neuroendocrine tumor improper for ligation with suction was resected en bloc by underwater endoscopic submucosal dissection

**DOI:** 10.1055/a-2183-6550

**Published:** 2023-10-27

**Authors:** Hirotaka Oura, Daisuke Murakami, Yasuki Hatayama, Harutoshi Sugiyama, Makoto Arai, Takayoshi Nishino

**Affiliations:** Department of Gastroenterology, Tokyo Womenʼs Medical University Yachiyo Medical Center, Chiba, Japan

For rectal neuroendocrine tumors (NETs) smaller than 10 mm, a meta-analysis indicated that endoscopic mucosal resection with suction, such as using a cap-fitted endoscope or ligating device, had a higher complete resection rate and significantly shorter procedure time compared to endoscopic submucosal dissection (ESD) 1 . In contrast, for NETs 10 to 14 mm in diameter that are improper for ligation with suction, ESD is feasible, although the treatment strategy has been controversial 2 . Herein, we report a case of a rectal NET that was successfully resected by underwater ESD (U-ESD) within a short time.

A man in his seventies underwent colonoscopy and was determined to have a slightly depressed submucosal tumor 10 mm in size in his lower rectum ( Fig. 1 ). The tumor was diagnosed as a NET histologically by biopsy. Endoscopic ultrasonography suggested the lesion was confined to the submucosa ( Fig. 2 ). Because suction was difficult due to the size of the lesion, the surgeon decided to resect it with U-ESD. The tumor was dissected in a layer just above the muscle layer and resected en bloc ( Fig. 3 , Fig. 4 ; Video 1 ). The time for resection lasted 8 minutes. The wound was completely closed with clips. Histological findings showed a NET G1 according to the World Health Organization classification with a negative margin ( Fig. 5 ). There was no evidence of vascular invasion. U-ESD enables submucosal dissection utilizing a floating effect in a magnified view 3 . In this case, the advantages of underwater conditions made it easy to proceed with the dissection at a depth just above the muscle layer, facilitating vertical margin negative excision. In conclusion, for lesions larger than approximately 10 mm, U-ESD can be a useful option for en bloc resection within a time that is comparable to endoscopic mucosal resection with suction.

**Fig. 1:**
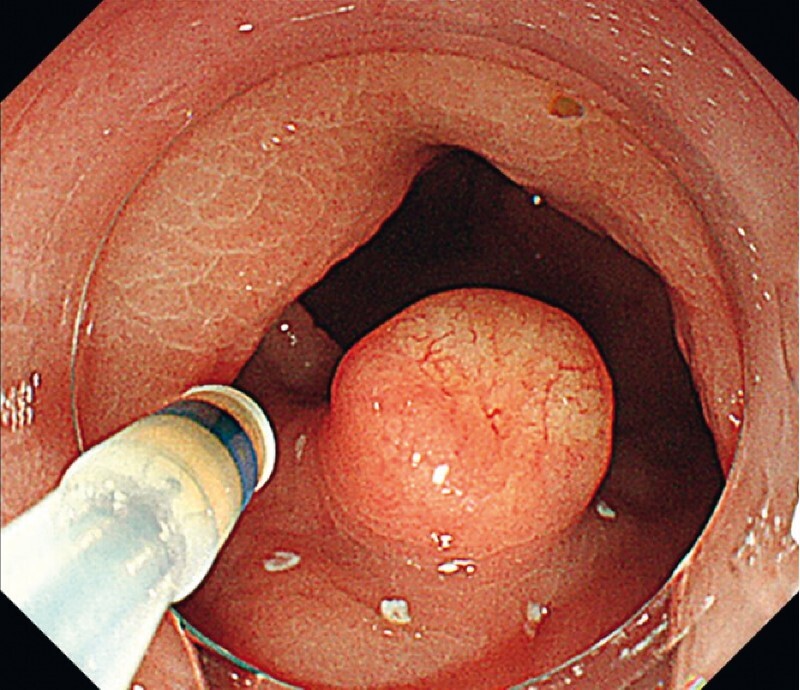
White light image before resection indicates a 10-mm yellowish, slightly depressed submucosal tumor located 2 cm from the anal verge in the lower rectum.

**Fig. 2:**
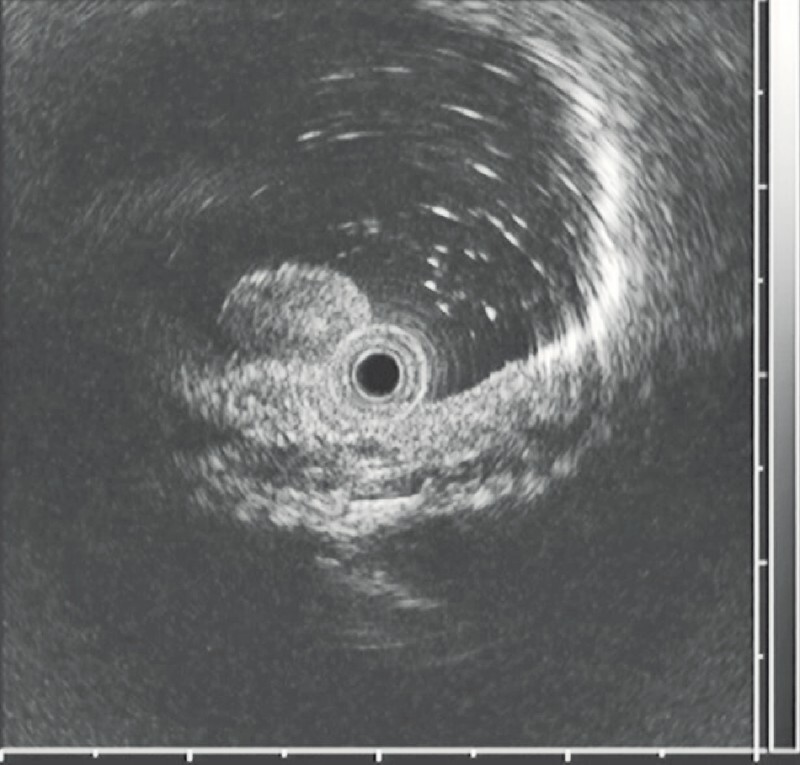
Endoscopic ultrasonography revealed the lesion was in the submucosa.

**Fig. 3:**
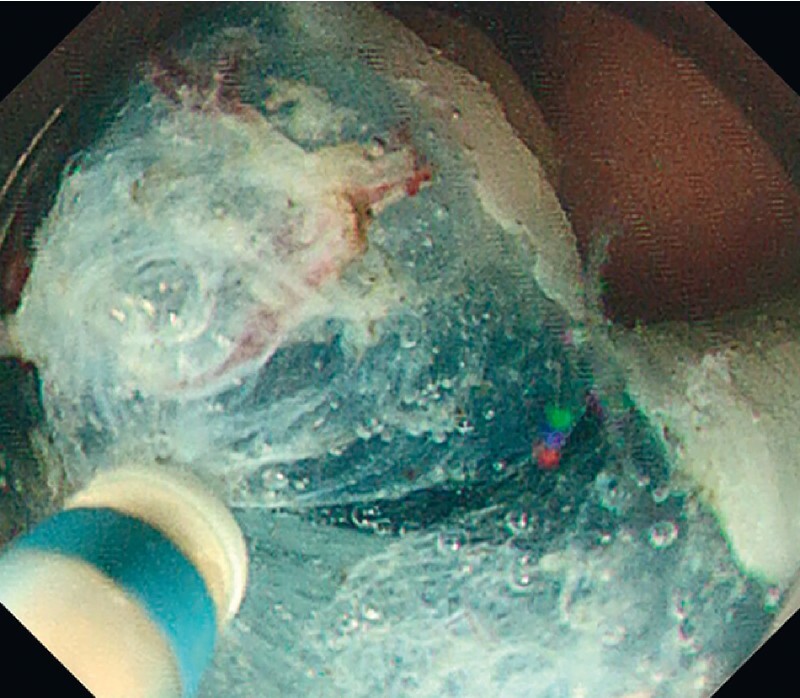
Submucosal dissection using a DualKnife J (KD-655Q; Olympus, Tokyo, Japan) just above the muscle layer was possible due to the floating effect in a magnified clear view in underwater conditions.

**Fig. 4:**
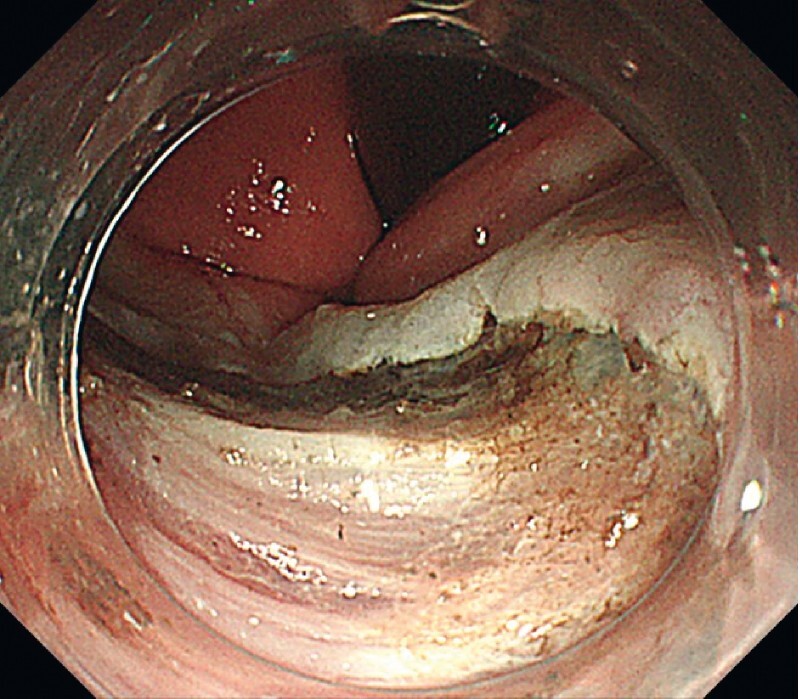
The tumor was resected in a layer just above the muscle layer.

**Video 1** Underwater endoscopic submucosal dissection for a neuroendocrine tumor in the lower rectum.


**Fig. 5:**
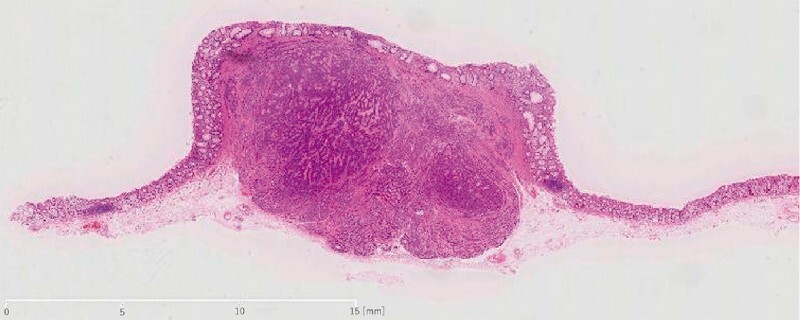
Histological findings of the tumor. The tumor was diagnosed as a neuroendocrine tumor G1 according to the World Health Organization classification, with a negative margin (hematoxylin–eosin staining).

Endoscopy_UCTN_Code_TTT_1AQ_2AD

## Notes

Endoscopy E-Videos

https://eref.thieme.de/e-videos
*E-Videos* is an open access online section of the journal *Endoscopy* , reporting on interesting cases and new techniques in gastroenterological endoscopy. All papers include a high-quality video and are published with a Creative Commons CC-BY license. Endoscopy E-Videos qualify for HINARI discounts and waivers and eligibility is automatically checked during the submission process. We grant 100% waivers to articles whose corresponding authors are based in Group A countries and 50% waivers to those who are based in Group B countries as classified by Research4Life (see: https://www.research4life.org/access/eligibility/ ). This section has its own submission website at https://mc.manuscriptcentral.com/e-videos
